# Supplementary material for: Incubator-based Sound Attenuation: Active Noise Control In A Simulated Clinical Environment
Source: PLoS One. 2020 Jul 15;15(7):e0235287. doi: 10.1371/journal.pone.0235287 (PMC7363066; doi:10.1371/journal.pone.0235287)
Supplement: Attachment — Submitted filename: Response Letter.docx [file pone.0235287.s001.docx]

May 23, 2020

*PLoS One*

Anne Lee Solevåg, M.D., Ph.D.
Academic Editor

Dear Dr. Solevåg,

We are happy to learn that PLOS ONE feels that our manuscript has merit and are appreciative of the opportunity to improve it in response to referee comments. We have responded to feedback point by point below:

ACADEMIC EDITOR:

1. All data files are available from [invictusmed.com/nicu_sim_data](http://invictusmed.com/nicu_sim_data)" - the link does not seem to work

- We have repaired the hyperlink and the data files are available. We apologize for the oversight.

2. Please make sure that the abbreviation NICU is being explained the first time it is mentioned

- Thank you, we have addressed this issue.

JOURNAL REQUIREMENTS

1. Please ensure that your manuscript meets PLOS ONE's style requirements, including those for file naming

- Thank you, we have ensured the manuscript meets style requirements.

2. We note that one or more of the authors is affiliated with the funding organization, indicating the funder may have had some role in the design, data collection, analysis or preparation of your manuscript for publication; in other words, the funder played an indirect role through the participation of the co-authors.

If the funding organization did not play a role in the study design, data collection and analysis, decision to publish, or preparation of the manuscript and only provided financial support in the form of authors' salaries and/or research materials, please review your statements relating to the author contributions, and ensure you have specifically and accurately indicated the role(s) that these authors had in your study in the Author Contributions section of the online submission form. Please make any necessary amendments directly within this section of the online submission form.  Please also update your Funding Statement to include the following statement: “The funder provided support in the form of salaries for authors [insert relevant initials], but did not have any additional role in the study design, data collection and analysis, decision to publish, or preparation of the manuscript. The specific roles of these authors are articulated in the ‘author contributions’ section.”

- We have included the requested statement.

The funder provided support in the form of salaries for authors [GH, LD], but did not have any additional role in the study design, data collection and analysis, decision to publish, or preparation of the manuscript. The specific roles of these authors are articulated in the ‘author contributions’ section.

One of the authors, Dr George Hutchinson is the Chief Executive Officer and the Chief Scientific Officer of the funder. Beyond what’s noted in the Author Contributions section, neither the board of directors nor the investors of the funder has had any influence on any aspect of this research.

3. Please also provide an updated Competing Interests Statement declaring this commercial affiliation along with any other relevant declarations relating to employment, consultancy, patents, products in development, or marketed products, etc.

Within your Competing Interests Statement, please confirm that this commercial affiliation does not alter your adherence to all PLOS ONE policies on sharing data and materials by including the following statement: "This does not alter our adherence to  PLOS ONE policies on sharing data and materials.” (as detailed online in our guide for authors http://journals.plos.org/plosone/s/competing-interests). If this adherence statement is not accurate and  there are restrictions on sharing of data and/or materials, please state these. Please note that we cannot proceed with consideration of your article until this information has been declared.

Please know it is PLOS ONE policy for corresponding authors to declare, on behalf of all authors, all potential competing interests for the purposes of transparency. PLOS defines a competing interest as anything that interferes with, or could reasonably be perceived as interfering with, the full and objective presentation, peer review, editorial decision-making, or publication of research or non-research articles submitted to one of the journals. Competing interests can be financial or non-financial, professional, or personal. Competing interests can arise in relationship to an organization or another person. Please follow this link to our website for more details on competing interests: <http://journals.plos.org/plosone/s/competing-interests>

- We have included the following Competing Interest Statement to declare the commercial affiliations of GH and LD.
- KA declares that no competing interests exist.
  GH has read the journal's policy and has the following competing interests:
  * is an employee, board member, and shareholder of the company manufacturing the equipment used in this study
  * is a named inventor of patent applications assigned to the company manufacturing the equipment used in this study.
  LD has read the journal's policy and has the following competing interests:
  * is an employee and shareholder of the company manufacturing the equipment used in this study
  * is a named inventor of patent applications assigned to the company manufacturing the equipment used in this study.

This does not alter our adherence to  PLOS ONE policies on sharing data and materials.

4. Please include a copy of Table 2 which you refer to in your text on page 4.

- We apologize for the confusion on this matter. The article has only one table. In the conversion from Word to PDF, the second reference to Table 1 was changed to “Table 2.” We will ensure the conversion is correct.

5. We note you have included a table to which you do not refer in the text of your manuscript. Please ensure that you refer to Table 1 in your text; if accepted, production will need this reference to link the reader to the Table.

- Please see the response to question 4. The manuscript only contains one table.

**REVIEWER #1:**

1. What was the Duty cycle? Could this be explained in more detail.

- Thank you for this important question. We have replaced “Duty Cycle” with “% time active” in Table 1. Duty cycle is an engineering description of the relative period that a signal is present within a given period of time. A duty cycle of 100% indicates that a signal is present throughout a time period while a duty cycle of 50% indicates that the signal is present half of the time. We believe that the new descriptor, “% time active” is more clear. Thank you for pointing out the use of jargon.

2. What statistical tests did you used?

- Thank you for this question. Our manuscript is largely descriptive and we did not perform statistical analysis.

3. How does the active noise canceling device work? More details would give the reader a clearer understanding about the device and how it works.

- We are appreciative of the opportunity to provide further clarity on how this device works. We have added the following text near line 82.

“Utilizing the phenomenon of incident waves summing, active noise control was accomplished by generating sound waves within the incubator that are out of phase with a model of sound waves detected by the outside noise sensor as they are when they have passed through the walls of the incubator. The residual noise remaining when the environmental and the cancelling sound wave meet was detected by the residual noise sensor, which is used to continually refine the cancelling sound wave produced to maximize the cancellation.”

4. Is this device approved by health authorities like the FDA?

- This device is subject to regulatory clearance by the FDA before clinical use may be permitted. However, as this study is preclinical (simulation), FDA clearance was not required.

5. If you use CPAP as white background noise, would you also register the same level of dB for alarms?

- We appreciate the chance to clarify this matter regarding combining a primary noise with a background noise. Measurements of the equivalent continuous sound level (Leq) of a primary noise and a background noise consider the contribution of all sounds during the measurement period. Background noise will increase the Leq over the measured time period.

Note that a background noise *can* affect the “detectability” of a primary tone. However, this is not reflected in the equivalent sound level, Leq. This would affect the Tone-to-noise-ratio and the Prominence ratio. However, these are not the subject of this study.

Regards,

Kaashif Ahmad, MD, MSc

Clinical Assistant Professor of Pediatrics, Baylor College of Medicine

Director of Research, Pediatrix Medical Group of San Antonio

Research Director of Neonatal Clinical Trials, MEDNAX National Medical Group
